# Effectiveness comparisons of Chinese patent medicine on treating premature ejaculation: A systematic review and meta-analysis

**DOI:** 10.1097/MD.0000000000017729

**Published:** 2019-11-01

**Authors:** Song Sun, Xuwei Mo, Yufeng Li, Ziqi Gong, Jisheng Wang, Xudong Yu, Liang Han, Hong Zhou

**Affiliations:** a Department of Surgery, Beijing Changping Hospital of Traditional Chinese Medicine; b Department of Andrology, Dongzhimen Hospital, Beijing; c Department of Andrology, Fangshan Hospital, Beijing University of Chinese Medicine, Chinan, China.

**Keywords:** Chinese patent medicine, premature ejaculation, systematic review

## Abstract

**Background:**

Premature ejaculation (PE) is a form of male sexual dysfunction. As people's lifestyle changes and the population ages, the incidence of PE continues to increase. Chinese patent medicines have been widely used in clinical practice as derivatives of traditional Chinese medicine (TCM). Many clinical trials have proven that Chinese patent medicine has a significant effect in the treatment of PE. In this systematic review, we aim to evaluate the effectiveness and safety of Chinese patent medicine for PE.

**Methods:**

We will search for PubMed, Cochrane Library, AMED, Embase, WorldSciNet, Nature, Science online and China Journal Full-text Database, China Biomedical Literature CD-ROM Database, and related randomized controlled trials included in the China Resources Database. The time is limited from the construction of the library to September 2019. We will use the criteria provided by Cochrane 5.1.0 for quality assessment and risk assessment of the included studies, and use the RevMan 5.3 and Stata 13.0 software for meta-analysis of the effectiveness, recurrence rate, and symptom scores of PE.

**Ethics and dissemination:**

This systematic review will evaluate the efficacy and safety of TCM for treating PE. Because all of the data used in this systematic review and meta-analysis have been published, this review does not require ethical approval. Furthermore, all data will be analyzed anonymously during the review process Trial.

**Trial registration number:**

PROSPERO CRD42017065316

## 1 Introduction

Premature ejaculation (PE) is a form of male sexual dysfunction. Definitions of PE consider the time to ejaculation, the inability to control or delay ejaculation, and the negative consequences of PE.^[1,2]^ One widely used definition is the persistent or recurrent ejaculation with minimal sexual stimulation before, on, or shortly after penetration and before the person wishes it. PE can be either lifelong (primary) or acquired (secondary). Lifelong PE is that which has been present since the person's 1st sexual experiences, while acquired PE is that which begins later following normal ejaculation experiences.^[3–5]^ PE may occur secondary to another condition such as erectile dysfunction or prostatitis. Men with PE are more likely to report lower levels of sexual functioning and satisfaction, and higher levels of personal distress and interpersonal difficulty than men without PE.^[6,7]^ They may also rate their overall quality of life lower than that of men without PE. In addition, the partner's satisfaction with the sexual relationship has been reported to decrease with increasing severity of the man's condition. Surveys in the United Kingdom, the United States, and other countries suggest that PE is the most common male sexual dysfunction, with prevalence rates of 18% to 31%.^[8–10]^ The treatment of PE should attempt to alleviate concern about the condition as well as increase sexual satisfaction for the patient and the partner. Treatments for PE mainly include drug therapy and psychologic and behavioral therapy.^[11]^ Oral 5-HT receptor reuptake inhibitors (SSRIs) are well-established and effective therapies for the treatment of PE, including fluoxetine,^[12]^ paroxetine,^[13]^ sertraline,^[14]^ dapoxetine,^[15]^ and so on. However, the side effects of SSRIs, such as nausea, vomiting, and dry mouth are somewhat confusing for clinicians.^[16]^ At the same time, evidence shows that the efficacy of psychologic and behavioral therapy is also not clear.

Chinese patent medicine is based on traditional Chinese medicine (TCM). Under the guidance of TCM theory, to prevent and treat diseases, it is processed into a certain dosage form of TCM products according to the prescribed prescription and preparation process, which is approved by the State Drug Administration. A class of TCM preparations. As the most important part of TCM, Chinese patent medicines have been widely used in clinical practice as derivatives of Chinese herbal medicine. TCM believes that the cause of PE is mainly in the kidney.^[17,18]^ Yin deficiency and heat, qi stagnation and blood stasis is its main pathogenic factor. Through the application of TCM in the treatment of PE's unique diagnosis and treatment system, clinical efficacy is significant.^[19,20]^ Modern research has shown that effective active ingredients in Chinese patent medicine can improve the blood supply of peripheral blood vessels and achieve therapeutic purposes.^[21]^ Through the action mechanism of multifaceted and multitarget, Chinese patent medicine regulates the body function as a whole and has unique advantages in the treatment of PE.

In the preliminary searches of the electronic databases, we found that randomized controlled trials (RCTs) of Chinese patent medicine for PE are on the rise.^[22,23]^ However, due to the limitation of the size and number of clinical centers, most clinical trials are small samples with low quality and lack of evidence-based exploration. Besides, the publication of the similar systematic review has not been retrieved in the database. Therefore, this review hopes to adopt meta-analysis to evaluate the efficacy and safety of Chinese patent medicine in the treatment of PE and provide evidence for its application in clinical practice.

## 2 Methods

This is a systematic review and ethical approval was not necessary.

### 2.1 Study registration

This systematic review protocol has been registered on PROSPERO as CRD42017065316 (http://www.crd.york.ac.uk/PROSPERO/display_record.php?ID=CRD42017065316). The protocol follows the Cochrane Handbook for Systematic Reviews of Interventions and the Preferred Reporting Items for Systematic Reviews and Meta-Analysis Protocol (PRISMA-P) statement guidelines. We will describe the changes in our full review if needed.

### 2.2 Eligibility criteria

#### 2.2.1 Type of study

Take Chinese patent medicine combined with other effective interventions as main treatment, including RCTs of the control group. Language is limited in Chinese and English. Non-RCTs, quasi-RCTs, case series, case reports, and crossover studies will be excluded.

#### 2.2.2 Participants

The cases included are adult male patients over 18 years old who have diagnosed PE. The region, nation, ethnic, and sources are not limited.

#### 2.2.3 Types of interventions

##### 2.2.3.1 Experimental interventions

Chinese patent medicines or the combined western medicine are used as experimental interventions. Other TCM treatments such as intravenous medication, acupuncture, and massage will be limited.

##### 2.2.3.2 Control interventions

As for the control interventions, who accepted simple western medicine can be used as a control intervention or did not get any treatment as a blank control would be adopted. However, once they had accepted the therapy of Chinese patent medicines or other TCM treatments, the trials will be rejected.

#### 2.2.4 Outcomes

##### 2.2.4.1 Primary outcomes

The primary outcome measurement will be IELT.

##### 2.2.4.2 Secondary outcomes

We also need to pay attention to the following outcomes: PE diagnostic tool, Arabic index of PE, and index of PE. More importantly, the adverse reactions of patients during medication will also be taken seriously.

#### 2.2.5 Data source

##### 2.2.5.1 Electronic searches

Database Search: PubMed, Cochrane Library, AMED, Embase, WorldSciNet, Nature, Science online and China Journal Full-text Database, China Biomedical studies CD-ROM Database, China Resources Database. A studies review of clinical studies on acupuncture for the treatment of PE was published in domestic and foreign biomedical journals from the establishment of the library to September 2019. Based on the standards of the Cochrane Collaboration Workbook of the International Evidence-Based Medicine Center, a manual and computer-based approach is used to conduct relevant studies searches. Search terms include: Chinese patent medicine, proprietary Chinese medicine, PE, and sexual dysfunction. The complete PubMed search strategy is summarized in Table 1.

**Table 1:**
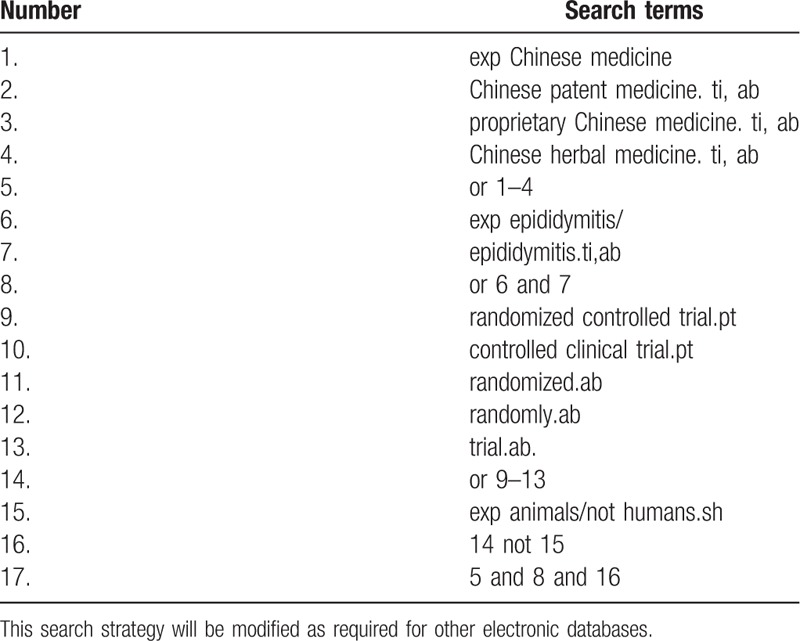
Search strategy used in PubMed database.

##### 2.2.5.2 Searching other resources

The manual search is mainly for dissertations, ongoing experiments, and gray literature. We will look for abstracts of dissertations, conference papers, and conference papers related to acupuncture and PE. Ongoing trials for the new reviews that are relevant to this term will be retrieved from the WHO International Clinical Trials Registry Platform, ClinicalTrials.gov, and the Chinese Clinical Trial Registry. For ongoing experiments, we will try to contact the trial author to help provide up-to-date clinical data. Potential gray literature will be elected in OpenGrey.eu website.

#### 2.2.6 Data collection and analysis

**Figure 1:**
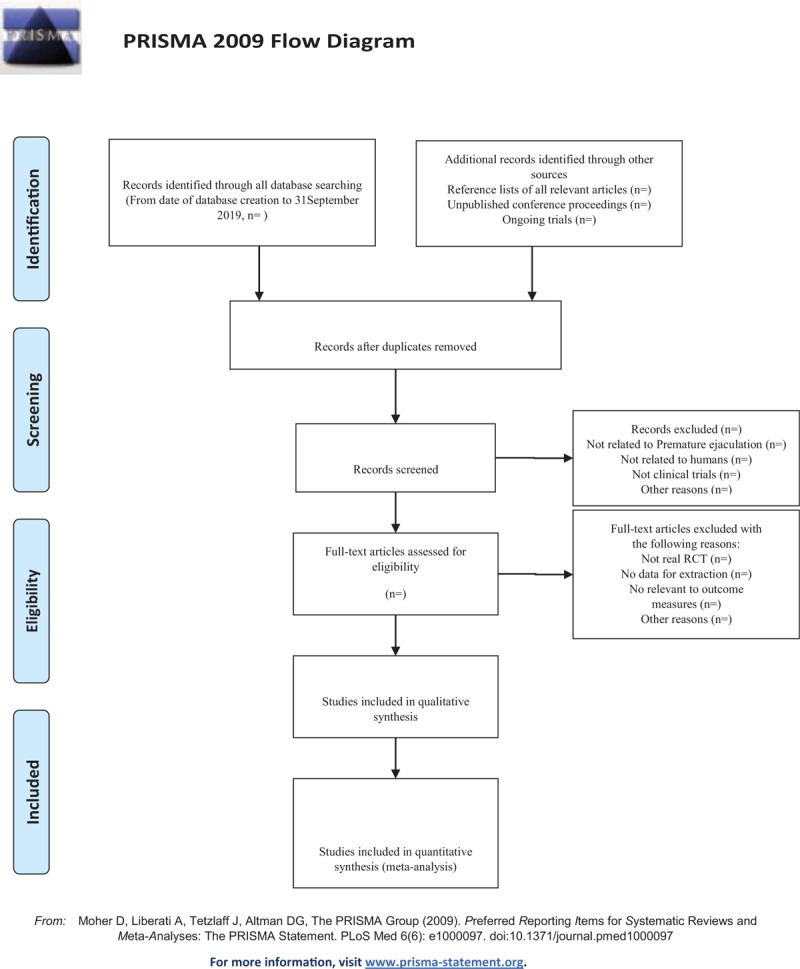
The Preferred Reporting Items for Systematic Reviews and Meta-Analysis (PRISMA) flow chart.

The EndnoteX7 software has been applied to manage the included references. Two qualified evaluators independently screened the titles and abstracts of the selected studies, excluding duplicates and documents that did not significantly conform to the study. After a preliminary evaluation, the selected documents will be read one by one. Exclusions were based on inclusion criteria for uncontrolled studies, no randomization, inconsistent assessment criteria, and similar data. If there are different opinions, the 3rd reviewer should be consulted. Studies information and data extraction were carried out on the final included studies, including the experimental methods of the study, the basic information of the included cases, the observation period, the intervention methods, observation indicators, and test results of the treatment group and the control group. The details of selection process will be shown in the PRISMA flow chart (Fig. 1).

#### 2.2.7 Risk of bias

The quality of the studies will be assessed by using the Cochrane Handbook 5.1.0. The assessment will include random sequence generation, randomization correctness, allocation scheme hiding, blinding of patients and implementers, accuracy of data results, and other risk of bias. The risk of low bias is expressed as “low risk” and the risk of high bias is expressed as “high risk.” The information provided in the studies is inaccurate or does not provide sufficient information for the bias assessment to be expressed as “unclear risk.” The earlier mentioned content evaluation was independently evaluated by 2 researchers, and any differences will be resolved through discussions with the 3rd reviewer.

#### 2.2.8 Statistical analysis

The meta-analysis in this study will use RevMan 5.3 and Stata 13.0 statistical software. Heterogeneity tests will be used for the included experimental studies. The numerical variable will be expressed as the normalized mean difference with a confidence interval of 95%. The heterogeneity of each pairwise comparison will be tested by Chi-squared test (test level *α* = 0.1). If there is no heterogeneity, a fixed effect model will be used. If there is significant heterogeneity between a set of studies, we will use a random effects model for meta-analysis. We will explore the reasons for the existence of heterogeneity from various aspects such as the characteristics of the subjects and the degree of variation of the interventions. The source of heterogeneity is further determined by means of sensitivity analysis.

#### 2.2.9 Publication bias

If a result of a meta-analysis contains more than 10 articles and above, we will use a funnel plot to test the risk of publication bias. Quantitative methods such as Begg testing and Egger testing will be used to help assess publication bias in the application.

#### 2.2.10 Quality of evidence

The grading of recommendations assessment, development, and evaluation (GRADE) method will be used to assess the quality of evidence for key outcomes. This assessment will be conducted through a guideline development tool (GRADEpro GDT, https://gradepro.org/).

## 3 Discussion

In recent years, with the changes in people's lifestyles and the aging of the population, the incidence of PE has increased.^[24]^ TCM believes that the etiology and pathogenesis of PE is related to spleen and kidney deficiency and qi and blood block. TCM can play the role of strengthening the spleen and kidney, promoting blood circulation and collaterals, and at the same time can improve the mood and achieve the purpose of treatment.^[25]^ From the modern medical point of view, some active ingredients in TCM can not only improve the blood supply of peripheral blood vessels, but also play a corresponding therapeutic role.^[26]^ Chinese patent medicine is based on TCM. Under the guidance of TCM theory, to prevent and treat diseases, it is processed into a certain dosage form of TCM products according to the prescribed prescription and preparation process, which is approved by the State Drug Administration. A class of TCM preparations. As the most important part of TCM, Chinese patent medicines have been widely used in clinical practice as derivatives of Chinese herbal medicine. With the deepening of understanding of PE, the trials and clinical reports of Chinese patent medicines for PE have gradually increased. Whether it is syndrome differentiation or special disease, Chinese patent medicines have achieved good results in the treatment of PE. To the best of our knowledge, there has been no comparison of the efficacy and safety of Chinese patent medicines in the treatment of PE in recent years. Therefore, we will compare the effectiveness and safety of Chinese patent medicines in the treatment of PE with systematic evaluation and meta-analysis. The results of this study can provide a possible ranking for the treatment of PE by Chinese patent medicines. We hope that the results will provide clinicians with the best options for treating PE and provide research directions. Although we will conduct a comprehensive search in this study, languages other than Chinese and English will be restricted, which will lead to some bias. In addition, the relevant literature on the treatment of PE in Chinese patent medicines is small and the overall quality is low, which may affect the authenticity of this study. Therefore, we hope that in the future, we will have a more rigorous and reasonable multicenter RCT to explore the clinical efficacy of Chinese patent medicines in the treatment of PE, so that the conclusion is more objective and reasonable.

## Author contributions

**Data curation:** Song Sun.

**Formal analysis:** Song Sun.

**Methodology:** Ziqi Gong.

**Project administration:** Xuwei Mo, Liang Han.

**Resources:** Ziqi Gong, Jisheng Wang, Xudong Yu.

**Software:** Xuwei Mo, Jisheng Wang, Xudong Yu, Liang Han.

**Supervision:** Yufeng Li, Xudong Yu, Liang Han, Hong Zhou.

**Validation:** Yufeng Li, Liang Han, Hong Zhou.
